# Synthesis of the Magnetically Nanoporous Organic Polymer Fe_3_O_4_@SiO_2_-NH_2_-COP and Its Application in the Determination of Sulfonamide Residues in Surface Water Surrounding a Cattle Farm

**DOI:** 10.1155/2022/6453609

**Published:** 2022-04-23

**Authors:** Yuqin Yang, Junjie Miao, Zhendong Yin, Weili Hao, Hongmei Shi, Ling Ma, Tiesheng Shi

**Affiliations:** ^1^Hebei Key Laboratory of Environment and Human Health, School of Public Health, Hebei Medical University, Shijiazhuang 050017, Hebei, China; ^2^Chemical Laboratory, Shijiazhuang Center for Disease Control and Prevention, Shijiazhuang 050011, Hebei, China; ^3^College of Chemistry, Chemical Engineering and Materials Science, Zaozhuang University, Zaozhuang 277160, Shandong, China

## Abstract

Efficient extractions of trace antibiotic residues in the environment are a key factor for accurate quantification of the residues. A new nanoporous material, namely, magnetically covalent organic polymer (MCOP, Fe_3_O_4_@SiO_2_-NH_2_-COP) was synthesized in this work and was used for magnetic solid-phase extraction (MSPE). The combination of MSPE with high-performance liquid chromatography separation together with ultraviolet detection (HPLC-UV) was established as an effective method for the determination of four sulfonamide (SA) residues in surface water surrounding a cattle farm. The synthesized magnetic material was characterized by SEM, TEM, FT-IR, magnetic properties measurement system (MPMS), and nitrogen gas porosimetry. The material possessed many attractive features, such as a unique microporous structure, a larger specific surface area (137.93 m^2^·g^−1^) than bare Fe_3_O_4_ (24.84 m^2^·g^−1^), high saturation magnetization (50.5 emu·g^−1^), open adsorption sites, and high stability. The influencing parameters, including pH, the used amount of MCOPs, the type of eluent, adsorption solution, and desorption time, were optimized. Under the optimized conditions, the method conferred good linearity ranges (*R*^2^ ≥ 0.9990), low detection limits (*S*/*N* = 3, LOD, 0.10–0.25 *μ*g·L^−1^), and satisfactory recoveries (79.7% to 92.2%). The enrichment factor (EF) for the four SAs was 34.13–38.86. The relative standard deviations of intraday (*n* = 5) and of interday (*n* = 3) were less than 4.8% and 8.9%, respectively. The equilibria between extraction and desorption for SAs could be reached within 150 s. The proposed method was sensitive and convenient for detecting SA residues in complex environmental matrices, and the successful application of the new MCOPs as an adsorbent was demonstrated.

## 1. Introduction

As a large group of synthetic antibiotics, sulfonamides (SAs) are commonly used in pharmaceutical and livestock industries and aquaculture [1, 2]. The broad antimicrobial spectrum, high efficiency, and relatively low cost of SAs have stimulated their ubiquitous utilization in veterinary practices for prophylactic and therapeutic purposes to ensure adequate productivity and disease management. According to statistics [3], China produces more than 200,000 tons of antibiotics per year, of which more than 80,000 tons are used in animal husbandry and aquaculture. Most veterinary antibiotics are not completely adsorbed in the animal intestines. The unabsorbed parts are transmitted into the environment in the form of prototypes and metabolites [4, 5] through runoff [6, 7], leaching, and manure used in the land [8], and eventually, they become the selection factor of resistant bacteria and multiple resistant bacteria [9]. Along with the overuse and abuse of antibiotics in recent years, antibiotic residues in the environment have caused continuous environmental pollution. Body exposure risks possibly produce some side effects for human beings, such as antibiotic resistance, allergic reactions, infectious diseases, and even a carcinogenic effect on living beings [10–12].

**Figure 1:**
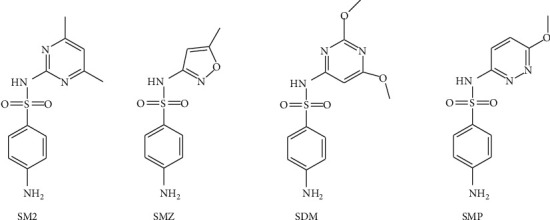
Chemical structures of SM2, SMZ, SDM, and SMP.

Sulfamethazine (SM2), sulfamethoxazole (SMZ), sulfadimethoxine (SDM), and sulfamethoxypyridazine (SMP) are the four most common SA drugs (their structures are given in Figure 1) because of their nature and water solubility. Although an increasing attention has been paid to the SA antibiotic residues in the environment in recent years, the low concentrations of SAs (typically at ppb levels) in environmental samples [13, 14] and the complex composition of the ecological matrix make it a very challenging task to determine these analytes. Thus, establishing a simple analytical method with high sensitivity for the determination of SA residues in the environment is highly demanding.

Various analytical methods for quantitating the SA residues in food and environmental samples have been reported [15–17], of which high-performance liquid chromatography (HPLC) coupled with different detectors was widely used. The ultraviolet (UV) detector has been the most commonly applied one for HPLC because of its price advantage [18]. Other sensitive detectors, such as fluorescence [19] and mass spectrometry (MS), [20, 21] have been applied to improve the detection sensitivity. These detectors are, however, relatively expensive for maintenance. Hence, the enrichment of the analytes by a sample pretreatment is a reliable approach to improve the sensitivity of determinations.

At present, liquid-liquid extraction (LLE) [22], liquid-liquid microextraction (LLME) [23], solid-phase extraction (SPE) [24], solid-phase microextraction (SPME) [25], and matrix dispersed solid-phase extraction (MDSPE) [26] are commonly used in the extractions and purifications of sulfonamides in samples. Among the solid-phase extractions, magnetic solid-phase extraction (MSPE) [27] has shown remarkable convenience. MSPE is based on magnetic or magnetizable materials. It has been reported that Fe_3_O_4_ magnetic nanoparticles (MNPs) can modify the functionally magnetic property of adsorbents, achieving high extraction efficiency for various targeting analytes [28]. Mesoporous carbon [29], carbon nanotubes [30, 31], graphene [32], metal-organic framework materials (MOFs) [33], and covalent organic framework materials (COFs) on the surfaces of magnets [34] have been prepared as functional magnetic adsorbents and have been explored and applied in MSPE for the capture of targeted analytes.

Materials modified by porous organic polymers (POPs) have attracted particular attention in recent years [35–38]. In general, POPs are composed of lighter elements, such as carbon, hydrogen, nitrogen, and oxygen, [39] and can also be classified as COFs [40–45], conjugated microporous organic polymers (CMPs) [46], polymers of intrinsic microporosity (PIMs) [47], hypercrosslinked polymers (HCPs) [48], and covalent porous organic polymers (COPs) [49]. In contrast with the good and highly ordered properties of COFs, which are synthesized from reversible reactions [50], COPs are highly crosslinked and amorphous. The structural diversity of COPs is attributed to the lack of crystal cell and orderly pore structures. The connection mode of the covalent bonds of COFs is relatively not very stable because of the unsaturated bonds involved (such as imine, hydrazine, etc.) [51]. COPs are connected contrarily by a rigid covalent C-C bond [52] and thus are very stable and not easy to be destroyed. The integrity of the structure of COPs can be maintained under harsh environmental conditions (for instance high temperature, strong acid, and strong alkali). Compared with MOFs or with COFs, COPs do not possess a crystal structure but give a high specific surface area and pore volume. Not surprisingly, the amorphous COPs have drawn a wide research interest [53].

The layers of COPs tend to aggregate in an aqueous solution, leading to a reduction in surface area and adsorption capacity. In addition, COPs are very light. They are difficult to recover from their suspensions and achieve high-throughput processing of a large number of samples. Magnetic cores can be introduced into the COPs dispersion process, which enables the rapid elution of the target analytes, and concurrently, the aggregations of COPs in the aqueous solution can be prevented. Moreover, the combination of the COPs with magnetic nanoparticles has enabled rapid adsorption and desorption equilibrium. Collectively, MSPE integrates multifunctionally nanoporous materials and magnetic nanoparticles (MNPs) and brings a number of superior advantages over conventional SPE sorbents [54, 55]. They can improve extraction efficiency by increasing the contacts between analytes and sorbents, facilitating the mass transfer efficiency of targeted analytes.

SAs contain amine groups and multiple sulfonic acid groups. Based on the nature of SAs, hydrophilic magnetic COPs (MCOP or Fe_3_O_4_@SiO_2_-NH_2_-COP) nano balls were designed and synthesized in this work and were applied to selectively recognize SAs. The amino-modified Fe_3_O_4_ was used as the magnetic core, and the network was synthesized by a controlled nucleophilic substitution of cyanuric chloride and 1,3,5-benzenetrithiol, forming low energy and chemically stable backbone structures [56]. As such, the sulfur bridged and nitrogen-rich nanoporously covalent organic polymers (COPs) were synthesized for the first time and utilized as an adsorbent for the extraction of the four SA residues.

## 2. Materials and Methods

### 2.1. Chemicals and Reagents

SM2 and SMZ were purchased from the National Institute for the Control of Pharmaceutical and Biological Products (NICPBP, Beijing, China). SDM was bought from Dr. Ehrenstorfer GmbH (Augsburg, Germany). SMP was obtained from Beijing Solarbio Science & Technology Co., Ltd (Beijing, China). The purity of all the above SAs was >98.0%. Methanol and acetonitrile were purchased from Thermo-Fisher (Pittsburgh, PA) and were of HPLC grade. N,N-diisopropylehtylamine (DIEA), 1,4-dioxane, 3-Aminopropyltriethoxysilane (APTES), and cyanuric chloride were obtained from Aladdin Biochemicals (Shanghai, China). Tetraethoxysilane (TEOS) and polyethylene glycol (PEG-4000) were bought from Shanghai Acmec Biochemicals. Ferric chloride hexahydrate (FeCl_3_∙6H_2_O), aqueous ammonia (NH_3_∙H_2_O), sodium hydroxide (NaOH), hydrochloric acid (HCl), sodium acetate anhydrous (NaAc), formic acid, absolute ethanol, and ethylene glycol were bought from Sinopharm (Shanghai, China). 1,3,5-benzenetrithiol was obtained from TCI Chemical Industrial Development Company (Shanghai, China). Acetone was purchased from Tianjin Oubokai Chemical Products (Tianjin, China). All the above reagents were analytically pure, except for acetone, which was chromatographically pure. Deionized ultrapure water with a specific resistance of 18.25 MΩ cm was used to prepare all the solutions.

The stock solutions of SAs (SM2, SMZ, SMP, and SDM) at a concentration of 1.0 mg·mL^−1^ were prepared in 0.10 mol·L^−1^ NaOH solution. Working standard solutions were prepared daily by an appropriate dilution of the stock solutions with water. All these solutions were stored in a refrigerator at 4°C.

### 2.2. Apparatus and HPLC-UV Conditions

SEM images were recorded on a cold field emission scanning electron microscope (S-4800, Hitachi, Japan). TEM images were recorded on a Tecnai G2 F20 field emission transmission electron microscope at 200 kV (FEI, USA). FT-IR spectra were recorded between 4000 and 500 cm^−1^ using an FT-IR-650 spectrometer (GANGDONG SCI&TECH, Tianjin, China). The magnetization curves were calculated by MPMS (XL-7, Quantum Design, USA), which used a superconducting quantum interference device magnetometer (SQUID). A Quadrasorb EVO (Quantachrome Instruments, USA) surface area and porosity analyzer was used to measure the nitrogen gas porosimetry and the Brunauer-Emmett-Teller (BET) surface areas after the samples were outgassed under vacuum at 100°C for 4 h.

**Figure 2:**
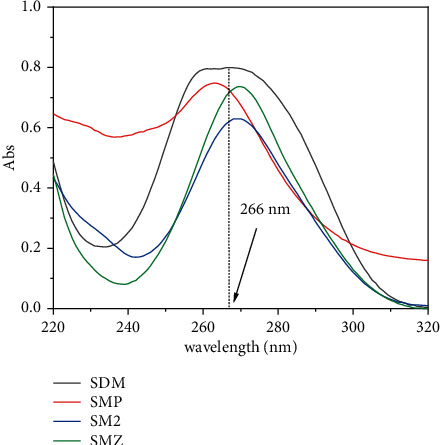
The UV-vis absorption spectra of the four SAs in aqueous solution.

The determination of SA residues was performed on an HPLC-UV (Ultimate 3000, Dionex, USA), and a Hypersil Gold C18 column (250 × 4.6 mm, id 5 *μ*m) was employed. Gradient elution was employed, and the mobile phases were acetonitrile (solvent A) and 0.1% formic acid aqueous solution (solvent B). A flow rate was set at 1.0 mL·min^−1^. The gradient elution was a 20-min process, encompassing the following changes: an initial gradient of 16% acetonitrile was held for 3 min, which was increased to 25% acetonitrile in 4 min and to 50% in 5.3 min. Then, it was increased to 90% in 1.7 min and held for 1.6 min, and finally, it returned to the initial mobile phase ratio in 6 min. The UV detection wavelength was chosen at 266 nm (cf. UV-vis spectra in Figure 2). The column temperature was maintained at 30°C, and the injection volume was 20.0 *μ*L.

### 2.3. Synthesis of Magnetic Adsorbents

#### 2.3.1. Synthesis of Fe_3_O_4_

Highly dispersed spherical MNPs of Fe_3_O_4_ were synthesized by the solvothermal method [57]. Firstly, 40 mL of ethylene glycol and 1.35 g of FeCl_3_∙6H_2_O were added to a 100 mL beaker to form a homogeneous and transparent yellow solution under vigorous magnetic stirring. Then 1.0 g of PEG-4000 and 3.6 g of NaAc were added to the solution sequentially under stirring to prevent particle aggregation. The solution mixture was stirred for about 30 min until the solution turned brownish-yellow and was then transferred into an autoclave lined with PTFE and kept at 200°C for 10 h for further reactions. Finally, the obtained magnetic nanoparticles were washed several times with ethanol and then dried in a vacuum oven at 60°C for 24 h.

#### 2.3.2. Synthesis of Fe_3_O_4_@SiO_2_

The MNPs of Fe_3_O_4_@SiO_2_ were synthesized by the sol-gel method with some modifications [58, 59]. Briefly, 150 mg of freshly prepared Fe_3_O_4_ MNPs and 80 mL of anhydrous ethanol were added to a 250 mL round-bottom flask, followed by the addition of 20 mL water (as the hydrolysis reaction medium). The mixture was sonicated for 15 min. Subsequently, 0.5 mL of aqueous ammonia was added, and the sonication was continued for 5 min. Then, 1.0 mL of TEOS was slowly and drop-wisely added to the reaction mixture under stirring at 30°C for 24 h. The obtained product was washed several times with water and dried at 60°C for 24 h.

#### 2.3.3. Synthesis of Fe_3_O_4_@SiO_2_-NH_2_

Firstly, 100 mg of Fe_3_O_4_@SiO_2_ MNPs and 30 mL of anhydrous ethanol were added to a 100 mL round-bottom flask, and the mixture was sonicated for 15 min to form a homogeneous one. Then, 1.0 mL of APTES and 20.0 mL of anhydrous ethanol were pipetted into the mixture, and the sonication was continued for 3 min. Next, the solution was stirred vigorously under reflux at 70°C for 12 h. The product was collected by an external magnet and was washed several times with anhydrous ethanol to remove residual impurities and dried under vacuum at 60°C for 24 h.

#### 2.3.4. Synthesis of Fe_3_O_4_@SiO_2_-NH_2_-COP

The synthesis was carried out according to a published procedure [60] with some modifications. 250 mg of Fe_3_O_4_@SiO_2_-NH_2_ was dispersed in 15.0 mL of 1,4-dioxane in a 250 mL round-bottom flask, and the mixture was sonicated for 20 min. The flask was then placed in an ice-water bath for a continuous stir under a nitrogen environment. After 5 min, 60.0 mL of 1,4-dioxane solution containing 0.26 g 1,3,5-benzenetrithiol and 15.0 mL 1,4-dioxane solution containing 0.25 g cyanuric chloride were added drop-wisely to the above solution, followed by the addition of 1.25 mL DIPEA. The mixture containing gray-black precipitates was stirred in an ice-water bath for 1.0 h, at 25°C for 2.0 h, and then at 85°C for 21.0 h. The gray-black precipitates were washed with 1,4-dioxane once and with ethanol thrice, and they were sonicated for 5 min and washed five times with water. Finally, the precipitates were dried under vacuum at 80°C for 12 h.

**Figure 3:**
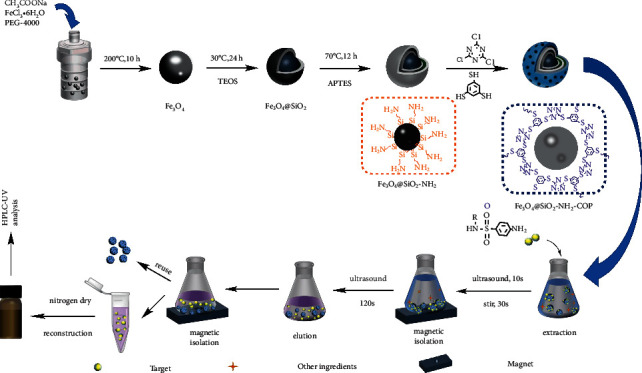
The synthetic route for Fe_3_O_4_@SiO_2_-NH_2_-COP, the structure of Fe_3_O_4_@SiO_2_-NH_2_-COP surface, and its application in the MSPE procedure.

The synthetic route for Fe_3_O_4_@SiO_2_-NH_2_-COP and the subsequent extraction process for the sulfonamides are illustrated diagrammatically in Figure 3.

### 2.4. Sample Preparations and MSPE Procedure

Sulfonamides are widely used in the prevention and treatment of infectious diseases in dairy cattle. Water samples were collected from a pond near a cattle farm in Shijiazhuang, Hebei Province of China (1000 mL at different sampling points and 0.5 m underneath the water surface). Five sampling points of the pond were chosen as the northeast (sample 1), northwest (sample 2), southeast (sample 3), southwest (sample 4), and the midway between the northeast and northwest corner (sample 5). The samples were put in light-tight glass bottles, and the sampling bottles were oscillated to ensure water uniformity. Samples were transported to the laboratory and stored at 4°C for later analysis. The samples were filtered through the membranes of 0.45 *μ*m, and the pH was then adjusted to 4.0.

Firstly, 9.0 mg of Fe_3_O_4_@SiO_2_-NH_2_-COP was dispersed in 20.0 mL of a sample solution, and the mixture was sonicated for 10 s to disperse the agglomerated material well, followed by a 30-second stirring on a shaker. Secondly, the absorbent was collected with an external magnet. The SAs were desorbed from the absorbent by a 2-min sonication with a 4.0 mL of desorbent (acetone solution containing 6% ammonia), and the desorbed solution was collected and evaporated to nearly dry by a nitrogen flow. The residues were then redissolved in 0.50 mL of the mobile phase at the initial gradient. The mixture solution was vortexed for 2 min and filtered through a membrane of 0.22 *μ*m, being ready for HPLC -UV analysis.

## 3. Results and Discussion

### 3.1. Characterization of the Magnetic Materials

**Figure 4:**
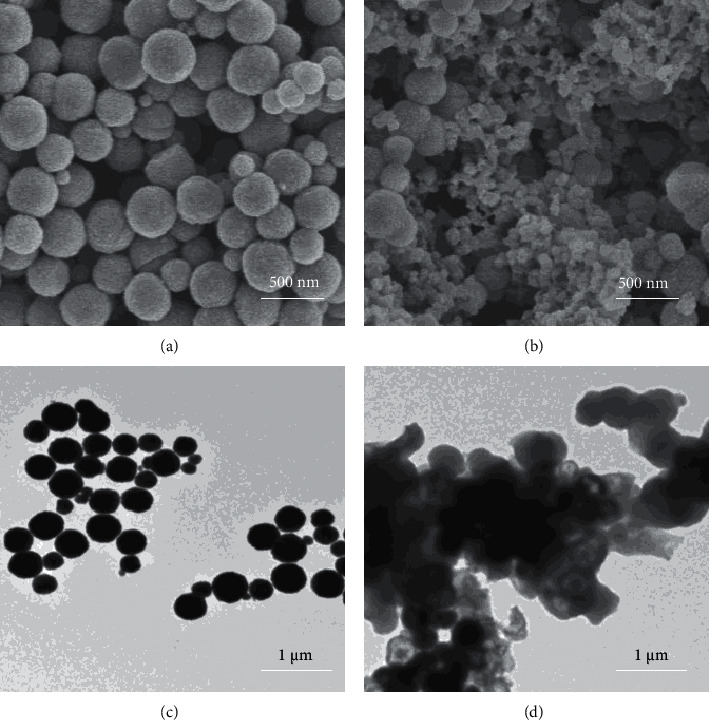
The SEM images of Fe_3_O_4_ (a) and Fe_3_O_4_@SiO_2_-NH_2_-COP (b). The TEM images of Fe_3_O_4_ (c) and Fe_3_O_4_@SiO_2_-NH_2_-COP (d).

The morphological structures of bare Fe_3_O_4_ and Fe_3_O_4_@SiO_2_-NH_2_-COP were characterized by SEM and TEM, as shown in Figure 4. Clearly, the well-dispersed Fe_3_O_4_ MNPs (figures 4(a) and 4(c)) were spherically shaped with a particle size of about 500 nm. When compared with the bare MNPs of Fe_3_O_4_, the surface of Fe_3_O_4_@SiO_2_-NH_2_-COP (figure 4(b)) was rougher. Fe_3_O_4_@SiO_2_-NH_2_-COP microspheres were composed of a compact core and a lower density shell. It had a uniform lamellar structure with numerous nanoflakes perpendicularly grafted to the surface of Fe_3_O_4_ MNPs. Moreover, a thinner layer was deposited on the surface of the Fe_3_O_4_ MNPs (Figure 4(d)). The thickness of the wrapped layer about 5 nm demonstrates the successful coating of the MCOPs.

**Figure 5:**
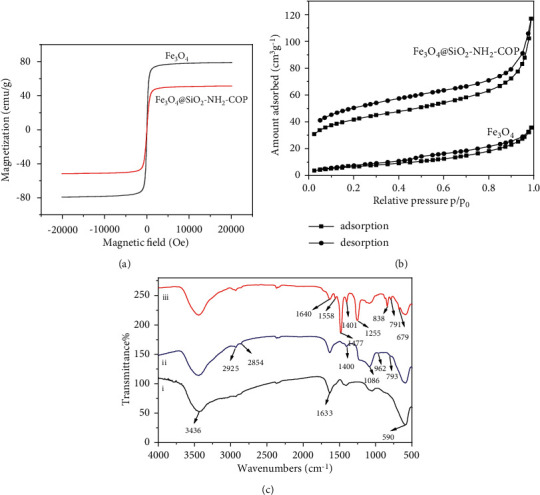
(a) Magnetization curves of Fe_3_O_4_ and Fe_3_O_4_@SiO_2_-NH_2_-COP. (b) N_2_ adsorption-desorption isotherms of Fe_3_O_4_ and Fe_3_O_4_@SiO_2_-NH_2_-COP. (c) FT-IR spectra of (i) Fe_3_O_4_, (ii) Fe_3_O_4_@SiO_2_-NH_2_, and (iii) Fe_3_O_4_@SiO_2_-NH_2_-COP.

Adequate magnetism is necessary to ensure a rapid separation of magnetic materials from liquid samples. MPMS analysis was used to assess the magnetic properties of synthetic materials.  Figure 5(a) illustrated the magnetic hysteresis loops measured at room temperature for Fe_3_O_4_ and Fe_3_O_4_@SiO_2_-NH_2_-COP, indicating that the two materials had similar sizes of the loops and exhibited zero coercivity and remanence. Thus, both were superparamagnetic. Moreover, the saturation magnetizations were 78.1 emu·g^−1^ and 50.5 emu·g^−1^, respectively. The lower saturation magnetization of Fe_3_O_4_@SiO_2_-NH_2_-COP could be the shielding effect of the silica shell on the surface of Fe_3_O_4_. However, the magnetic response was sufficient for magnetic separation in practical applications.

As shown in figure 5(b), the nitrogen gas porosimetry provided direct proof for the successful synthesis of the Fe_3_O_4_@SiO_2_-NH_2_-COP. The bare Fe_3_O_4_ MNPs possessed a type II adsorption isotherm with a typically nonporous nature. However, Fe_3_O_4_@SiO_2_-NH_2_-COP showed a type I isotherm, indicating its unique microporous structure. Furthermore, the BET surface area of Fe_3_O_4_@SiO_2_-NH_2_-COP was calculated to be 137.93 m^2^g^−1^, which was much higher than that of the bare Fe_3_O_4_ MNPs (24.84 m^2^·g ^−1^). The pore volume and pore width of Fe_3_O_4_@SiO_2_-NH_2_-COP are 0.142 cm3g^−1^ and 1.614 nm seperately, which were calculated by density functional theory (DFT) [61]. The high specific surface area and porosity of the adsorbent provided a sufficiently high loading capacity.

Some characteristic bonds were identified for Fe_3_O_4_, Fe_3_O_4_@SiO_2_, Fe_3_O_4_@SiO_2_-NH_2_, and Fe_3_O_4_@SiO_2_-NH_2_-COP by FT-IR spectra. All the spectra in figure 5(c) show a sharp absorption peak around 590 cm^−1^, which was assigned to the Fe-O vibration of Fe_3_O_4_. All materials showed broad bands around 3436 cm^−1^ and 1633 cm^−1^, which were attributed to the stretching and bending vibrations of water molecules on the surface of Fe_3_O_4_. Spectrum (ii) in figure 5(c) demonstrates that Fe_3_O_4_@SiO_2_-NH_2_ gave a new broadband absorption at 1086 cm^−1^ and a weak band at 962 cm^−1^, which corresponded to the vibrations of Si-O-Si and Si-O-Fe, respectively. Furthermore, the peaks around 2925 cm^−1^ and 2854 cm^−1^ were attributed to the C-H asymmetric and symmetric stretching vibrations in the aminopropyl group from APTES molecules. In addition, the peak around 1400 cm^−1^ was related to the stretching vibration of C-N from APTES molecules. All these absorption bands indicate that the amorphous SiO_2_-NH_2_ was coated successfully on the surface of Fe_3_O_4_.

It was shown by spectrum (iii) in figure 5(c) that several strong bands in the region of 1200–1600 cm^−1^ corresponded likely to the typical stretching of C-N heterocycles in Fe_3_O_4_@SiO_2_-NH_2_-COP. Additionally, the characteristic breathing mode of the triazine units is evident at around 800 cm^−1^. The absence of the C-Cl stretching at 850 cm^−1^ confirms that the three chlorides on cyanuric chloride were all substituted. Moreover, the C-S bond at 791 cm^−1^ in Fe_3_O_4_@SiO_2_-NH_2_-COP is stronger than that in 1,3,5-benzenetriol (751 cm^−1^). The absorption band around 1600–1200 cm^−1^ for COP from Fe_3_O_4_@SiO_2_-NH_2_-COP was designated to the vibrations of the aromatic ring skeleton. Two characteristic absorption bands at 1640 cm^−1^ (C=N) and 791 cm^−1^ (C-S) were attributed to the triazine rings in the network. Thus, it is concluded that COP was grafted successfully on the Fe_3_O_4_@SiO_2_-NH_2_ surface.

### 3.2. Adsorption Condition

#### 3.2.1. Optimization of Adsorbent Amount

**Figure 6:**
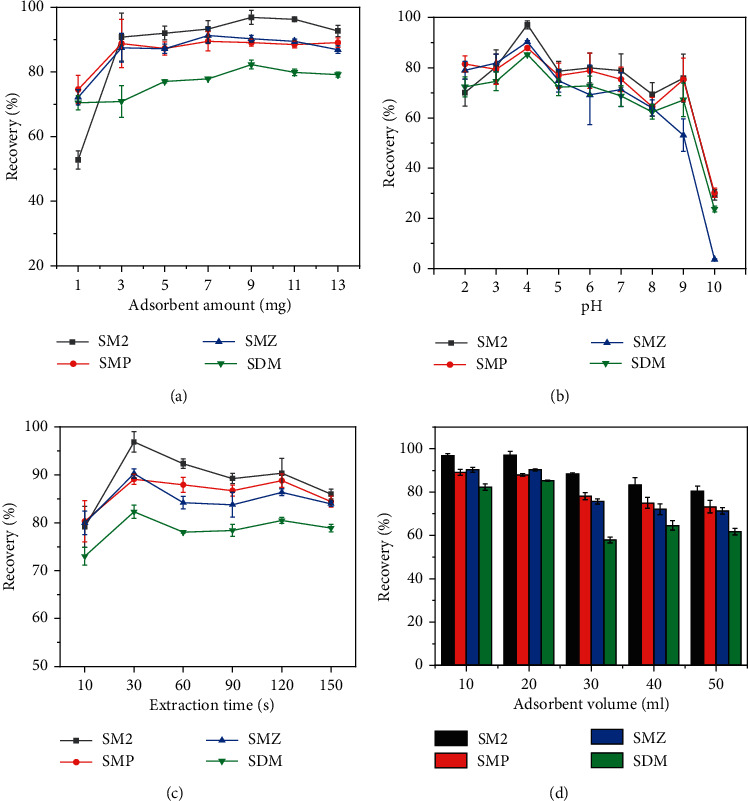
Recovery influence by the amount of Fe_3_O_4_@SiO_2_-NH_2_-COP (a), by pH (b), by extraction time (c), and by extraction volume (d). The concentrations of the analytes were 5.0 *μ*g·L^−1^.

In MSPE processes, the amount of adsorbent affects the extraction efficiency significantly. Generally, increasing the amount of adsorbent can provide more adsorption sites. In this work, different dosages of Fe_3_O_4_@SiO_2_-NH_2_-COP were evaluated, ranging from 1.0 mg to 13.0 mg. It is shown in figure 6(a) that when the amount of Fe_3_O_4_@SiO_2_-NH_2_-COP was increased from 1.0 to 9.0 mg, the recoveries of analytes also increased. However, a further increase virtually did not improve the recoveries. Therefore, 9.0 mg of Fe_3_O_4_@SiO_2_-NH_2_-COP was selected as the optimum amount.

#### 3.2.2. Optimization of Solution pH

The pH was a key factor affecting the extraction of SAs by Fe_3_O_4_@SiO_2_-NH_2_-COP. It is because of the fact that the protolysis species of SAs vary with the pH of the solution. Sulfonamides are amphoteric compounds with two functional groups (aromatic amine and sulfonamide amide), which can accept and donate protons, respectively [62, 63]. The proton transfers between the protonation and deprotonation groups, depending on the solution pH. As shown in Table 1, the four SAs possess two deprotonation sites, with p*K*_a1_ = 1.74–2.26 for the aromatic amines and p*K*_a2_ = 5.81–7.45 for the sulfonamide amides [64]. There exist three protolytic species (cationic, neutral, or anionic forms) relying on the solution pH for each of the four SAs. When the pH of the solution is below p*K*_a2_, the SAs molecules are protonated and positively charged, and when the pH is greater than p*K*_a2_, the compound gives the proton and is negatively charged. Moreover, Fe_3_O_4_@SiO_2_-NH_2_-COP consisted of a large *π*-electron system of melamine and 1,3,5-benzenetrithiol. At pH 4.0, SAs in the mixture are in the cationic form and are more readily captured by the sorbent. Therefore, at the sample solution pH 4.0, it is speculated that the electrostatic repulsion between the target analytes and the sorbent in the subsequent eluent system is the lowest, resulting in the highest final extraction recovery [65]. Since the phenyl group of sulfonamides is highly hydrophobic, the *π*-*π* interactions and hydrophobic forces should dominate the interaction between the analyte and the sorbent during elution. Therefore, the effect of pH variation across the solution system on the final extraction recovery was investigated in the range of 2.0 to 10.0. The recovery-pH curves for the four SAs are given in figure 6(b) and show a similar trend. The final extraction efficiency was the highest when the pH was 4.0, which was chosen as the optimum pH.

#### 3.2.3. Optimization of Extraction Time

The extraction time was studied between 10 and 150 s. In figure 6(c), the absolute recoveries of the four analytes were increased as the shaking time was increased from 10 s to 30 s, while a slight decrease was observed when the adsorption time exceeded 30 s. This phenomenon can be attributed to the possibility that the adsorption and desorption were in equilibrium, and a long shaking time was not favorable for the adsorption [62]. Sufficient interactions between the target analytes and the adsorbent can be achieved by a shaking time of 30 s. Therefore, a shaking time of 30 s was chosen as the adsorption time.

#### 3.2.4. Optimization of Extraction Volume

The water sample volumes were evaluated between 10.0 mL and 50.0 mL.  Figure 6(d) shows the recoveries of the four SAs gradually increased when the sample volume was changed from 10.0 mL to 20.0 mL. No additional enhancement was found when the sample volume was varied from 20.0 mL to 50.0 mL. Thus, the sample volume was selected as a volume of 20.0 mL.

### 3.3. Desorption Condition

#### 3.3.1. Optimization of the Type of Elution

**Figure 7:**
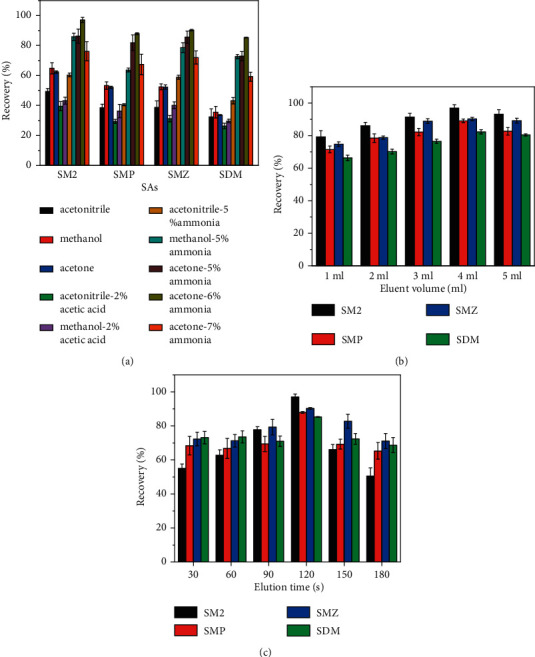
Effects of the elution types (a), eluent volumes (b), and elution times (c) on the desorption efficiency of SAs. The concentrations of analytes were 5.0 *μ*g·L^−1^.

To adequately elute the target analytes from the adsorbent and to avoid the interference of impurities, the desorption solvent must have a much higher affinity to the targeting analytes than to the sorbent. In this work, various common desorption solvents were analyzed, including acetonitrile, methanol, acetone, acetonitrile/ammonia, acetonitrile/acetic acid, methanol/acetic acid, methanol/ammonia, and acetone/ammonia. The recoveries of the four SAs by the acetone/ammonia mixture gave the best results. Subsequently, the acetone: ammonia ratios were investigated.  Figure 7(a) clearly shows that increasing the ammonia fraction could significantly improve the desorption efficiency. It was reasoned that since the pH value of the adsorbed solution was acidic, the addition of a certain amount of ammonia restored the solution to neutral, and the SAs were dislodged more readily from the pores of the material during sonication.  Figure 7(a) demonstrates that all the SAs could be recovered quantitatively when the ratio reached 94 : 6, which was chosen as the desorption solvent.

#### 3.3.2. Optimization of Eluent Volume

The volume of the eluent was also optimized, and the results are displayed in figure 7(b). The elution of the analytes increased with increasing eluent volume and reached a maximum when the eluent volume was 4.0 mL. Further increasing the eluent volume did not improve significantly the recoveries of the SAs, suggesting that 4.0 mL was enough to elute the SAs from the adsorbent. The optimal volume was thus determined to be 4.0 mL.

#### 3.3.3. Optimization of Elution Time

Different elution times (30, 60, 90, 120, 150, and 180 s) were chosen to extract the four targeting SAs. As shown in figure 7(c), the recoveries increased with the increase of elution time and reached the equilibria after 120 s for all SAs. By this time, more than 80% of the SAs were recovered. Since the distribution of analytes between absorbents and sample solution was in a dynamic equilibrium, the desorbed analytes may be adsorbed again. Therefore, an elution time of 120 s was selected for further optimizations.

### 3.4. Adsorption Mechanism

The MNPs of Fe_3_O_4_ can be easily destroyed by dissolution because of their intolerance to acids and bases, and thus the extraction efficacy was much poorer compared to Fe_3_O_4_@SiO_2_-NH_2_-COP. Therefore, the sulfur COPs played a key role in the extraction process. Firstly, a large number of sulfur-pair and nitrogen-pair electrons in the pores of Fe_3_O_4_@SiO_2_-NH_2_-COP could form intermolecular hydrogen bonds with amino and sulfonic groups in SAs [66]. Cyanuric chloride and 1,3,5-benzenetrithiol in Fe_3_O_4_@SiO_2_-NH_2_-COP form a large *π*-electron system. The p-empty orbits of N or S atoms in SAs can form p-*π* interactions with the *π*-electron system via electrostatic interaction. Hence, weak interactions, such as electrostatic attraction and hydrogen bonding, play an important role in the extraction process. Finally, Fe_3_O_4_@SiO_2_-NH_2_-COP has a high specific surface area and porosity, providing sufficient contact space and transfer channels for targeting SAs. Under such circumstances, the molecular screening effect can be avoided, rendering faster adsorption kinetics than other porous adsorbents.

### 3.5. Development of Analytical Method Based on the Adsorbent Fe_3_O_4_@SiO_2_-NH_2_-COP

#### 3.5.1. Effect of the Different Batches of Fe_3_O_4_@SiO_2_-NH_2_-COP

To ensure the reproducibility of Fe_3_O_4_@SiO_2_-NH_2_-COP between synthetic batches, the reaction temperature of each step should be strictly controlled [60]. Three different batches of Fe_3_O_4_@SiO_2_-NH_2_-COP were prepared separately and used as MSPE sorbents for the extraction of SAs.  Table 2 showed that the extraction recoveries of the analytes did not vary significantly, and the RSDs' % ranged from 0.7% to 4.6%, indicating that the synthesis of Fe_3_O_4_@SiO_2_-NH_2_-COP was virtually reproducible.

#### 3.5.2. Reusability of the Magnetic Adsorbent

**Figure 8:**
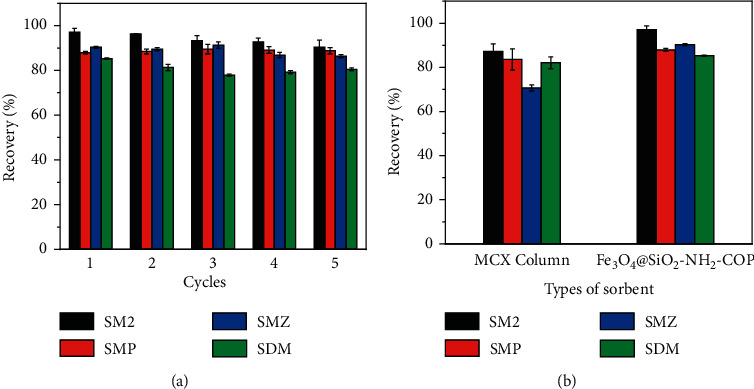
Reusing times of Fe_3_O_4_@SiO_2_-NH_2_-COP (a) and a comparison between Fe_3_O_4_@SiO_2_-NH_2_-COP and MCX-based SPE column on extraction efficiency (b). The concentrations of analytes were 5.0 *μ*g·L^−1^.

The used Fe_3_O_4_@SiO_2_-NH_2_-COP was treated by washing with methanol and acetone and was employed repeatedly in the subsequent MSPE processes. The recovery data in figure 8(a) indicates that the adsorbent could be reused at least five times for the extraction of SAs without significant degradations.

#### 3.5.3. Comparison with a Traditional Packed SPE Cartridge

The absolute recoveries from Fe_3_O_4_@SiO_2_-NH_2_-COP extractions were compared to those from an MCX sorbent of the traditionally packed SPE cartridge (National Standard of China GB 29694–2013). The obtained results are displayed on the right side of figure 8(b). No doubt, the absolute recoveries from Fe_3_O_4_@SiO_2_-NH_2_-COP extractions were significantly higher. Moreover, the magnetic COP-based MSPE method outperformed the MCX-based SPE one in the adsorbent dosage, time consumption, and operational efficiency.

#### 3.5.4. Method Validation

To evaluate the performance of the proposed Fe_3_O_4_@SiO_2_-NH_2_-COP-based MSPE, coupled with HPLC-UV, the linearity, the limit of detection (LOD), the limit of quantification (LOQ), and accuracy were examined under the above set of optimized conditions. In HPLC-UV quantification, a matrix-matched method, which did not contain SAs in the water samples (sample 3), was used to prepare calibration curves.  Table 3 shows that the method exhibited good linearity over a wide concentration range from 0.45 to 250.00 *μ*g·L^−1^ (with *R*^2^ = 0.9990 to 0.9995). LOD and LOQ were determined at the signal-to-noise ratios (*S*/*N*) of 3 and 10 times, respectively. The LODs were determined to be in the range of 0.10–0.25 *μ*g·L^−1^ and LOQs were in the region of 0.45–0.75 *μ*g·L^−1^, indicating that the proposed method is highly sensitive.

#### 3.5.5. Sample Analysis

**Figure 9:**
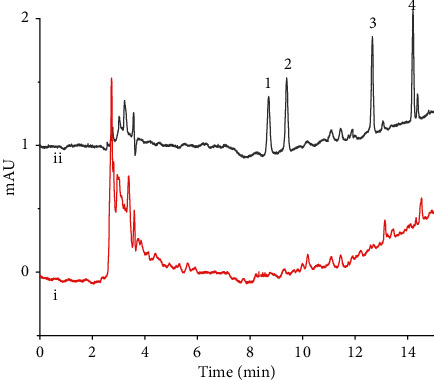
Chromatograms obtained for the water sample by the proposed method. (i) The sample. (ii) The sample spiked with the four SAs, 50.0 *μ*g.L^−1^. Peak assignments: 1, SM2; 2, SMP; 3, SMZ; 4, SDM.

As is shown in Table 4, the four SAs were not detected in any of the samples from the pond.  Figure 9 shows the chromatograms of the water sample by the proposed method. According to the National Standard of China GB 29694–2013, the MCX column was also used for solid-phase extraction to determine the four SAs. Again, none of the SAs was detected in the water samples. Possible explanations are that the concentrations of the SA residues in the pond were lower than the detection limits. Alternatively, these antibiotic residues were already decomposed.

#### 3.5.6. Recovery Experiments

To investigate the reliability of the proposed method, the recoveries of the four SAs were examined by adding the standard solutions to blank samples. The recoveries were found to be in a range from 79.7% to 92.2% (Table 5). In addition, the intraday and interday precisions were examined by injecting samples spiked with the four SAs at 50.0, 100.0, and 200.0 *μ*g·L^−1^ and by recording the peak areas. Precisions were observed to be in a range from 1.9% to 4.8% for intraday measurements and from 3.4% to 8.9% for the interday assays.

#### 3.5.7. Comparison with Other Methods

The methods for the extractions of four SAs in terms of sorbent dosage, LOD, enrichment factor, time of MSPE, and relative recoveries are summarized in Table 6 for comparison [62, 67–73]. The conventional dispersive solid-phase extraction (dSPE) requires an additional centrifugal step and is cumbersome to perform. The adsorption efficiency of the present method was superior to most of the reported sorbents. Moreover, the losses during MSPE were limited and less time-consuming, conferring that Fe_3_O_4_@SiO_2_-NH_2_-COP is a good MSPE for the fast and efficient analysis of SAs in water samples.

To further evaluate the extraction efficiency, the enrichment factor (EF) was introduced and calculated according to the following equation: EF = *C*_1_/*C*_0_, where *C*_1_ and *C*_0_ represent the analyte concentration in the final solution and the analyte concentration in the initial sample solution, respectively. The values were derived to be 38.86 for SM2, 35.14 for SMP, 36.13 for SMZ, and 34.13 for SDM. By the enrichment of the sample solution, the limit of detection of the instrument was broken through and a more satisfactory sensitivity was obtained.

## 4. Conclusions

The use of SAs in animal feedings is not prohibited in many countries, thus SA residues in animal-derived foods and environmental media are possible, albeit at low levels. In this work, the new adsorbent Fe_3_O_4_@SiO_2_-NH_2_-COP was firstly designed and synthesized, and it was applied for the extraction of trace amounts of SAs in surface water surrounding a cattle farm, followed by HPLC-UV analysis. The sample concentrating step is faster than some classic methods, such as SPE, and the sample manipulation is minimal and is thus a lower-cost procedure. The proposed method confers relatively low detection limits, and thus an excellent sensitivity, although the enrichment factor is not very high. When the new Fe_3_O_4_@SiO_2_-NH_2_-COP as MSPE was combined with classical HPLC analysis, forming the analytical method developed in the work, the method has potential applications in monitoring the levels of antibiotic residues and organic pollutants in environmental media. Such magnetic materials in the field of separation provide an excellent option.

## Figures and Tables

**Table 1:** Four SA target compounds and their basic information.

Name	Abbreviation	Mass (g mol^−1^)	p*K*_a1_^a^	p*K*_a2_^b^	Reference
Sulfamethazine	SM2	278.33	2.26	7.45	[64]
Sulfamethoxypyridazine	SMP	280.30	2.08	7.19	[64]
Sulfamethoxazole	SMZ	253.28	1.74	5.81	[64]
Sulfadimethoxine	SDM	310.33	1.84	6.21	[64]

^a^p*K*_a1_ denotes the dissociation constant of the aromatic amino group. ^b^p*K*_a2_ is the dissociation constant of the sulfanilamido group.

**Table 2:** The precision between batches.

Analytes	Batches recovery%			RSD% (*n* = 3)
	Batch 1	Batch 2	Batch 3	
SM2	97.1 ± 1.6	91.3 ± 3.1	90.4 ± 2.7	3.9
SMP	87.9 ± 2.1	87.4 ± 0.6	86.7 ± 4.2	0.7
SMZ	90.3 ± 0.5	86.4 ± 2.4	89.4 ± 2.5	2.3
SDM	85.3 ± 0.3	78.5 ± 4.7	79.2 ± 0.8	4.6

**Table 3:** Regression parameters of the four SA analytes.

Analytes	Regression equation	*R* ^2^	LOD (*μ*g·L^−1^)	LOQ (*μ*g·L^−1^)	Linear range (*μ*g·L^−1^)
SM2	*Y* = 0.0012*X* + 0.0134	0.9990	0.25	0.75	(0.75–250.00)
SMP	*Y* = 0.0017*X* + 0.0887	0.9992	0.10	0.45	(0.45–250.00)
SMZ	*Y* = 0.0014*X* − 0.0045	0.9995	0.25	0.75	(0.75–250.00)
SDM	*Y* = 0.0012*X* + 0.0110	0.9994	0.10	0.45	(0.45–250.00)

**Table 4:** The analytical results of four SAs in real water samples.

Analytes	Sample 1	Sample 2	Sample 3	Sample 4	Sample 5
SM2	n.d.^a^	n.d.	n.d.	n.d.	n.d.
SMP	n.d.	n.d.	n.d.	n.d.	n.d.
SMZ	n.d.	n.d.	n.d.	n.d.	n.d.
SDM	n.d.	n.d.	n.d.	n.d.	n.d.

^a^Not detected.

**Table 5:** Precisions and recoveries for the determination of SAs in water samples.

Analytes	Intraday precision (RSD%, *n* = 5)			Interday precision (RSD%, *n* = 3)			Recovery (%, *n* = 3)		
	50.0 (*μ*g·L^−1^)	100.0 (*μ*g·L^−1^)	200.0 (*μ*g·L^−1^)	50.0 (*μ*g·L^−1^)	100.0 (*μ*g·L^−1^)	200.0 (*μ*g·L^−1^)	50.0 (*μ*g·L^−1^)	100.0 (*μ*g·L^−1^)	200.0 (*μ*g·L^−1^)
SM2	2.1	3.2	2.8	4.9	4.3	5.6	83.7 ± 1.9	81.4 ± 2.7	86.7 ± 2.9
SMP	3.0	4.0	1.9	5.5	6.2	7.7	84.2 ± 2.9	85.2 ± 4.4	79.7 ± 2.0
SMZ	3.9	4.8	2.1	3.8	8.9	4.6	81.1 ± 3.5	87.7 ± 4.8	80.9 ± 1.9
SDM	4.8	3.5	4.0	5.8	7.1	3.4	92.2 ± 3.8	89.8 ± 3.1	84.8 ± 3.4

**Table 6:** A comparison of various methods for the determination of SAs.

Method	Adsorbent	Matrix	Amount of sorbent (mg)	MSPE time (min)	LOD (*μ*g/L)	Recovery (%)	EF	References
MSPE-HPLC-AD^a^	HCP/Fe_3_O_4_^b^	Water	20.0	15.0	0.60–1.00	84.0–97.0	—	[67]
MSPE-UPLC-MS/MS	MIL - 101(Cr)@GO	Milk	5.0	50.0	0.012–0.145	79.8–103.8	10.00	[68]
MSPE-UPLC-MS/MS	CMGO^c^	Water	15.0	21.0	0.0005–0.0016	83.2–109.2	1320.00–1720.00	[62]
dSPE-UPLC-DAD	MMWCNT^d^	Water	300.0	— ^g^	3.36–6.90	22.0–77.0	—	[69]
MSPE-HPLC-UV	CoFe_2_O_4_-G^e^	Milk	15.0	22.0	1.16–1.59	62.0–104.3	—	[70]
SPE-HPLC-MS/MS	MSdt-MIPs^f^	Water	120.0	16.0	0.003–0.005	62.2–91.1	500.00	[71]
MSPE-LC-MS/MS	Fe_3_O_4_@MoS_2_	Water	6.0	25.0	0.0001–0.001	80.2–108.6	—	[72]
MSPE-HPLC-DAD	Fe_3_O_4_-GO	Water	5.0	30.0	50.0–100	67.4–119.9	—	[73]
MSPE -HPLC -UV	Fe_3_O_4_@SiO_2_-NH_2_-COP	Water	9.0	2.5	0.10–0.25	79.7–92.2	34.13–38.86	This work

^a^AD means amperometric detector. ^b^Magnetic hypercrosslinked polystyrene. ^c^Carboxylated magnetic graphene oxide nanoparticles. ^d^Magnetic multiwalled carbon nanotubes. ^e^Graphene-based magnetic nanocomposite. ^f^Magnetic surface double-template molecularly imprinted polymers. ^g^Not given in the text.

## Data Availability

The data used to support the findings of this study are included within the article.
